# Conflicting forces in the implementation of medicinal cannabis regulation in Uruguay

**DOI:** 10.1186/s42238-023-00189-6

**Published:** 2023-07-12

**Authors:** Eliana Alvarez, Rosario Queirolo, Belen Sotto

**Affiliations:** Department of Social Sciences, Universidad Católica del Uruguay, Montevideo, Uruguay

**Keywords:** Medicinal cannabis, Challenges, Uruguay

## Abstract

**Background:**

Uruguay is widely known as a pioneer country regarding cannabis regulation policies, as it was the first state to regulate the cannabis market for both recreational and medicinal purposes in 2013. However, not all aspects of the regulation have moved forward at the same speed. Medicinal uses keep facing several challenges that undermine patients’ effective access to treatments and products. What are those persistent challenges for the medicinal cannabis policy in Uruguay? This paper aims to describe and understand the current state of medicinal cannabis in the country and identify the most critical challenges and conflicting forces for its proper implementation.

**Methods:**

To do so, we conduct twelve in-depth interviews with key informants, including governments officials, activists, entrepreneurs, researchers, and doctors. These interviews are complemented with information from the congressional committees’ public records and other documentary sources.

**Results:**

This research shows that the legal framework was thought to assure quality products over access. The main challenges of medicinal cannabis in Uruguay are related to three issues: (i) the timid development of the industry, (ii) a limited and expensive supply, and (iii) the emergence of an informal productive sector.

**Conclusions:**

The political decisions regarding medicinal cannabis made in the last seven years have derived from a halfway policy that fails to guarantee patient access or promote the growth of a vibrant national industry. Positively, the several actors involved are aware of the extent of these challenges and new decisions have been made to overcome them, meaning that monitoring the future of the policy is very much needed.

**Supplementary Information:**

The online version contains supplementary material available at 10.1186/s42238-023-00189-6.

## Introduction

Uruguay is well known for its cannabis policy. In 2013, it became the first country in the world to fully regulate the cannabis market. the regulation’s origins and main characteristics are distinctively different than other reforms carried out worldwide (Queirolo 2020). One of the most relevant distinctions is that the recreational and medicinal uses of cannabis were regulated simultaneously, something rare in the international context. Typically, governments have first designed policies related to medicinal uses to later advance on recreational ones. For example, Canada and many states in the USA have followed this path (Caulkins et al. 2015) or have approved laws exclusively for medicinal uses, while recreational use continues to be illegal, such is the case of Israel, Colombia, and recently Argentina. In Uruguay, the same law (19.172) abolishes penalizations for activities related to the elaboration of medicinal or pharmaceutical products and authorizes recreational use under three legal access mechanisms (Robaina and Bardazano 2020).

Nevertheless, this common origin did not translate into the same at the time of implementing each regulation. In fact, while the legal acquisition of recreational cannabis is fully ongoing, users who want to acquire for therapeutic purposes still face significant challenges. This paper aims to study these challenges and, therefore, to understand how this policy is evolving in Uruguay.

In the last decades, many countries have decriminalized or approved policies to regulate medicinal cannabis. Therefore, a diversity of regulatory schemes has emerged worldwide, implementing multiple strategies to promote and govern the production, commercialization and prescription (Belackova et al. 2017; Rogeberg et al. 2018). On the one hand, some countries privileged the industry and broad accessibility to patients. To do so, they created flexible regimes even when this might facilitate the appearance of uncontrolled quality products. Examples of these reforms have taken place in Canada, Germany, the Netherlands, and several US states (Aguilar et al. 2018). On the other hand, others countries, like the Czech Republic, have designed strict policies for the elaboration and commercialization of cannabis-based medicines in order to guarantee quality. The side effect of these demanding policies was the implementation of a bureaucratic system that nourishes the emergence of a growing illicit supply channel (Schlag 2020).

Latin America has also experienced a medicinal cannabis regulation “boom” in recent years. The most known case might be Colombia (Decree 2.467/2015 and Law 1.787/2016), which regularized the use and trade of cannabis for medicinal purposes in 2016 and has experienced a great development of the industry (Calderón Vallejo et al. 2017; Rivera 2019). Other countries that have regulated or decriminalized production are Chile (Law 20.000, Decree 84/2015), Brazil (RDCs ANVISA/MS 17/2015 and 66/2016, Law 5.295/2019), Peru (Law 30.681/2017), Ecuador (Amendment of article 220 COIP/2019), Argentina (Law 27.350/2017), and Paraguay (Law 6.007/2017). Uruguayan medicinal cannabis regulation was passed at the same time than recreational cannabis; however, the implementation of the regulation concerning medicinal cannabis came later than the recreational one.

By conducting in-depth interviews with key informants, this paper aims to understand the main challenges and conflicting forces that medicinal cannabis policy faces after 7 years of its approval. It is organized as follows: the first section focuses on the legal path of medicinal cannabis in Uruguay. The second one describes the regulation scheme of the country. The third one details the methodology, and the fourth one enumerates the main challenges identified, divided in three main topics: industry, accessibility, and informal producers. Finally, we close with a general discussion about the implications of these findings for the medicinal cannabis regulation in Uruguay.

## The path of medicinal cannabis in the Uruguayan regulation

Law 19.172, approved in December of 2013, regulates the cannabis market in Uruguay for all its purposes (Asamblea General 2013). Article 5 depanelizes cannabis-related activities, including the elaboration of products for medicinal use (numeral A) or the investigation and industrialization for pharmaceutical use (numeral D). The law also created the Cannabis Cultivation and Regulation Institute (IRCCA) as the authority to implement and enforce the policy.

Specific regulations came later. In 2015, Decree 46/015 (Presidencia de la República 2015) approved the elaboration of “plant products”―herbs or a mixture of herbs for medicinal purposes―and “pharmaceutical specialties” for medicinal use. It also indicated that, for the elaboration of either of the two specialties, the authorization of IRCCA is mandatory, as well as the endorsement of the Ministry of Public Health (in Spanish MSP) following national regulations regarding the production of any medicament. A year later, Decree 403/016 (Presidencia de la República 2014) regulated the production and commercialization of “vegetable specialties” of any kind; in 2017, the professional prescription of cannabis-based drugs was authorized.

More recently, law 19.847 issued in 2020 (Camara de Senadores 2019) summarizes the previous legislation in four types of medicinal cannabis products: (1) pharmaceutical specialties approved by the Health Ministry (medicines in the traditional sense); (2)“vegetable” products (classified in (a) vegetable specialty, (b) new phytotherapeutic medication, (c) traditional phytotherapeutic medicine, and (d) plant product based on cannabis); (3) magistral preparations prescribed by physicians and elaborated by a pharmaceutical chemists, produced with cannabis extracts or standardized cannabinoids; and (4) foreign cannabinoid-based products for medicinal purposes imported with a physician’s prescription. Even though this law was approved by the Parliament, it has not been regulated by the Executive Power yet. The regulation of the law is responsibility of the Executive Branch and implies ensuring that it is implemented, the way it is implemented, and how it must be enforced. The lack of regulation means that several of the articles of the law have not been applied.

Finally, in July 2021, the national government promoted a new decree that repeals 46/015 and proposes a new legal framework for the medicinal cannabis industry (Decree 246/021). Among its novelties, it includes the possibility of producing feedstocks or semi-finished products and exporting them, which was banned so far only the export of finished products was allowed). Before this decree, companies needed to go through a strict registration process at the MSP to export their products. This dictate was controversial among business owners. With the new norm, that paperwork is no longer necessary for exportation. They still need authorization, but it is a less complex procedure (Ferrere 2021).

The regulatory path that the country has navigated is still under review. The decree that was recently signed, by which relevant previous norms are revoked, is a clear example. The need to improve the normative is a sign that the policy has not fulfilled its objectives yet. The Uruguayan legalization purpose was to guarantee public health by controlling quality and access mechanisms for cannabis users (Law 19.172). As we will see, the normative decisions have direct consequences in the persistent challenges identified in this paper.

## The Uruguayan regulation scheme

Worldwide, the regulatory framework adopted by countries tends to differ in a diversity of aspects. First, countries regulate the supply side of medicinal cannabis differently, for example, by indicating which authorized products can be produced, who can produce them, what compositions are allowed, and how they are commercialized, among other things (Belackova et al. 2017). As mentioned, some countries have designed more flexible policies to encourage production, while others have adopted strict mechanisms to ensure quality (Schlag 2020). In this distinction, Uruguay clearly belongs to the second group. The institutional actors involved in the licensing process, described above, indicate that the policy’s primary goal is to provide quality-controlled products.

Regulations might also be on the demand side by stipulating limits to patients’ characteristics, medical conditions for which cannabis is authorized, defining a list of professionals authorized to prescribe cannabis, and establishing the mechanisms of access. In fact, there is a limited list of diseases in Israel and the Czech Republic for which cannabis can be prescribed (Belackova et al. 2017; Coitiño et al. 2019). In Israel, physicians must fulfill some requirements like being authorized by a national agency; while in the Czech Republic, the prescription of cannabis depends on the medical condition that each physicians’ specialty is allowed to treat. In Colombia and some US states, medicinal cannabis can be accessed in different kinds of stores (pharmacies, dispensaries, homeopathy stores, etc.) (Coitiño et al. 2019). In Canada, patients or caregivers can cultivate themselves, with a medical authorization that specifies a daily quantity of cannabis to be used, buy it online or through dispensaries (Wadsworth et al. 2022).

Regarding these regulations, Uruguay also appears as a strict example. Despite any physician can prescribe cannabis through an authorized medical prescription and there are no restrictions to specific medical conditions, the only legal ways to access medicinal cannabis are either with a doctor’s prescription at pharmacies or importing it with a special authorization.

Finally, the extent of the state participation is something to pay attention to. This broadly refers to the existence of specialized government agencies, licensing mechanisms, quality checks, price policies, etc. Some countries, like Israel or the Netherlands, have created special agencies responsible for the licensing. Others rely on already existing authorities like health or narcotics institutions. Some cases have rigid production procedures, like the Czech Republic, while others, like Canada or some US states, leave bigger space to the market (Belackova et al. 2017). The Uruguayan regime is very state-centered. The licensing process for medicinal cannabis industries involves various state’ agencies: IRCCA, MSP, and the Ministry of Livestock, Agriculture and Fishing (MGAP in Spanish). These agencies provide different permissions depending on the purpose of the production (research, cultivation, elaboration) (Isoardi 2020). All cannabis-based medicines must be approved by the Department of Medicines of the MSP before their commercialization at pharmacies.

In sum, Uruguay’s medicinal cannabis policy is quality-oriented, but there is a lot still to be defined in terms of procedures for authorizing medicinal products. Moreover, how the regulation would be ultimately implemented will impact on patients’ access to drugs and treatments, and the growth of the cannabis industry.

## Methods

Aiming to identify and understand the persistent challenges of the medicinal cannabis policy in Uruguay, we conducted twelve in-depth interviews with key informants, including past and present authorities in charge of the implementation of the policy (3 interviews), physicians (2 interviews), representatives of patients and producers’ associations and activists (2 interviews), representatives of the industry and consultants (3 interviews), and researchers (2 interviews). Due to the pandemic, interviews were carried out virtually between February and July of 2021. Information is presented in an aggregated way to protect participants’ identities. The same guide was used for all interviews, with a particular thematic focus depending on the participants’ background (see Additional file 1). Responses were organized in four topics: national legislation, patients and accessibility, industry development, and informal producers.

We complemented our analysis with information provided in relevant documents and press releases on medicinal cannabis. Particularly, the acts of the Congress’ committees that studied the medicinal cannabis policy were very representative of the main discussion. The Health and Social Assistance Committee and the Special Committee on Addictions addressed the issue of medicinal cannabis in 2016 and 2017 respectively. Later, the Law 19.847 bill was discussed in the House, Health and Social Assistance Committee (from May to September 2019) and in the Senate Public Health Committee (from November to December 2019). The discussions carried out during seven sessions are public records available on the Congress’ website. Participants’ names and affiliations are stated in those records. Translations are our own.

## Results: main challenges of the medicinal cannabis policy

Based on the interviews and the revision of documentary sources, we identified three main challenges of the medicinal cannabis policy: the timid development of the industry, the lack of accessibility for patients, and the emergence of an unregulated market.

### The industry: timid development

Progress in the medicinal cannabis industry has been timid. Although the number of licenses granted for productive activities has grown consistently since 2017, IRCCA has authorized a total of nineteen licenses for industrialization (IRCCA 2021), many of which are not related to medicinal products (see Table 1). The most significant growth has been since 2020. This is consistent with the strategy of the new national government to promote the sector, as it has been identified with a great potential for the economic development of the country (Uruguay XXI 2020; Redacción 180 2021). Furthermore, in 2020, three companies were licensed to cultivate psychoactive cannabis for medicinal use and seven for industrialization or elaboration of pharmaceutical supplies (see Table 1).

**Table 1:** Industrialization licenses granted by IRCCA by purpose (2017–2020)

Purpose	2017	2018	2019	2020
Elaboration of CBD oil for a period of 3 (three) years.			1	
Elaboration of active pharmaceutical inputs full spectrum resin				1
Elaboration cosmetic products with non-psychoactive cannabis seed oil			1	
Plant product (Yerba)		1		
Extraction and manufacture of CBD oil		1		
Manufacture of products for human and veterinary use	1	1	1	1
Import of CBD, elaboration and commercialization of derived products			1	
Industrialization of cannabis for medicinal and cosmetic purposes				1
Industrialization of cannabis for medicinal purposes				5
Obtaining raw extract of psychoactive cannabis for medicinal use			1	
Processing of plant material to obtain cannabis extract			1	
**Total**	1	3	6	8

Source: Own elaboration based on information from the IRCCA

The interviewees described the procedure for obtaining a license for productive activities as complex. Central tensions are perceived at the institutional level between the different agencies directly involved in granting the licenses, partly because, besides the authorizations issued by IRCCA, obtaining a license requires other permits from the MGAP and/or the MSP (depending on whether the activity is cultivation, manufacturing, processing, etc.).

Although the IRCCA (IRCCA 2020) and the national government have taken steps to promote the sector (Poder Ejecutivo 2021), several interviewees highlighted the differences between the institutions views on the topic and how they impact on the industry. On one side, stakeholders described the bureaucratic procedures of the health authority (MSP) as cumbersome and stricter. According to Marco Algorta, president of the Chamber of Medicinal Cannabis Companies: “What we need is a regulatory framework that allows us to develop the industry. Today we are stuck because we face a dilemma that I do not know if any other industry has: we have many offers from foreign buyers, but the national barriers are the ones that prevent us from selling” (La Republica 2021). On the other side, the MGAP is pointed like an industry enhancer because of the precision of its requirements and the speed of its decisions. Many endeavors cultivate under MGAP permissions, even though they will need a MSP license to commercialize their products. In short, as Zeballos et al. (2020) concludes, so far, the bureaucratic mechanisms established for the implementation and supervision of the cannabis policy operate as disincentives for the growth of the local industry.

The most recent political decisions somehow try to solve these tensions. In 2020, two decrees were signed to permit―exceptionally―the exportation of hemp or cannabis for medicinal purposes that were cultivated between 2018-2020 (Decree 214/2020 and Decree 215/2020), without the specific authorization issued by the MSP, as it was usually required. This change was made permanent in the decree approved in July 2021, which eliminates certain institutional overlaps and allows the exportation of feedstocks without a license from the MSP. The normative reflects the intentions of the new pro-market government, led by president Lacalle Pou, to encourage and expand cannabis industry in the country (Uruguay XXI 2020).

### Accessibility: limited and expensive supply

The timid performance of the industry has direct effects on the quality and diversity of the legal medicinal cannabis market. This jeopardizes patients’ accessibility to products and treatments. At the time this research was conducted, the local market for cannabis-based drugs is quite scarce. To start with, the offer is limited to only three drugs authorized by the MSP (Epifractan, Xannadiol, and Xalex). The acquisition of all of them require a professional prescription.

Furthermore, these drugs are mainly prescribed for children with treatment-resistant epilepsy. Therefore, the local legal market excludes adults and most medical conditions since no other cannabis-based drugs are available. A member of an association of patients with fibromyalgia, Claudia Souto, states the problem in this way: “the option that the health system and the legal framework give us today does not solve our health or economic problems; it is not suitable with the reality. What is offered today in pharmacies is not what we need” (Comisión de Salud Pública y Asistencia Social 2019).

In addition, the legal market only offers CBD-based products. The ones named before have a CBD concentration that varies between 2.5 and 10%. This is because the regulation only allows the commercialization of non-psychoactive cannabis products (THC under 1%). The absence of medicines with higher THC concentrations is identified as another barrier to accessibility. It was emphasized by the physicians who participated in the discussion of Law 19.847: “We must have access to different types of cannabis products because now there is only one drug with two concentrations―2% and 5%―of cannabidiol, and we actually need THC” (Comisión de Salud Pública y Asistencia Social 2019). The consequences of this threshold are relevant for treating a diversity of conditions, like chronic pain or spasticity related to multiple sclerosis, for which THC has proven to be effective (Levinsohn and Hill 2020).

Another relevant aspect of cannabis-based medicines is the high price. The cost of the products available at pharmacies can vary between 30 and 170 dollars, making them almost unaffordable for patients who require frequent doses. Products could be less expensive for users if they were offered through the Health System . However, this is not the case in Uruguay. Authorized cannabis-based products have not been included in the Medicaments Therapeutic Form (in Spanish FTM), the national list of approved drugs to be considered by health providers’ pharmacopeias. The inclusion of these products in the FTM has been stated as one of the main goals of the recently implemented Medicinal Cannabis National Program (Facil Desviarse 2021), but until now, patients acquire them at pharmacies with a physician’s indication. A representative of the association of children with the West syndrome, Elizabeth Olivera (Fundación Batar), explain this difficulty: “Acquiring Epifractan is difficult for us (...) This month we are fine because we have the Christmas bonus; other times we relied on the help of family and friends. It seems to me that this is not the idea. We need the drug to be endorsed, to be granted by health institutions” (Comisión de Salud Pública y Asistencia Social 2019).

An alternative path is the Mechanism for the Import of Non-Regulated Products or “mechanism of compassionate use,” which consists of importing medicines available in other countries, which is an alternative to domestic supply that is sometimes used. Access through this way requires following a particular bureaucratic procedure and having a specific medical prescription known as an “orange prescription.” It can have a high cost depending on the medication indicated. Julia Galzerano, a physician member of the Uruguayan Society of Endocannabinology, referred to it in the Deputies committee: “you have to fill out a form and physicians have to make a special prescription (...) then you have to contact the suppliers. This implies time and money” (Comision Especial de Adicciones 2017). Unfortunately, authorities have interrupted this mechanism, and authorities are not receiving any new applications by patients.

Finally, when asked about how to improve accessibility, many key informants pointed to the magistral preparations as a middle ground solution between prescribed drugs and general herb products. This alternative exists in countries like Colombia, the Netherlands, Germany, and Switzerland (Cubillos-Sánchez 2021; Abuhasira et al. 2018). The possibility of elaborating magistral preparations based on cannabis is stated in Law 19.847 of 2020. The normative indicates that it would be manufactured according to each patient’s conditions and present the cannabinoid ratio indicated by the physician. Pharmaceutical chemists would oversee its elaboration, especially done in an authorized pharmaceutical establishment. However, this mechanism is not in effect yet.

Additional challenges emerged regarding the implementation of this alternative. First, it requires in-depth knowledge about cannabinoids and their properties to treat medical conditions from both physicians and chemists. Recent evidence from the Uruguay’s medical community shows that physicians’ self-perceived knowledge on cannabinoid-based products and the endocannabinoid’s system is low (Queirolo et al. 2021). Chemists are technically prepared to manufacture these preparations, but it is uncertain how much they know about cannabinoid-based products. Second, the regulation specifies that magistral preparations must be compounded in authorized pharmacies. The establishments should have the space, infrastructure, and human resources needed. So far, the actual capacity of pharmacies to develop this activity is under question. A representative of the chemists’ professional association said to the deputies in 2019: “We have the technical capacity to compound magistral preparations. [But] The reality is that today, the presence of a pharmaceutical chemist in pharmacies is not much (...) The presence of a chemist has been limited to perform balances of psychotropic drugs” (Comisión de Salud Pública y Asistencia Social 2019).

### Informal producers: the unregulated alternative

In the absence of plural and affordable legal options in the market, a growing informal productive sector has emerged, mainly integrated by individual small-scale producers and groups organized in civil associations. The latter has become the only option left for many of those who need to use cannabis for medicinal purposes. According to an exploratory study conducted on people who participated in the 2015 and 2016 editions of the ExpoCannabis, 42% of the patients had accessed products supplied by local third parties; these products were mainly oils (44%), creams (15%), and flowers (13%) (Peyraube et al. 2017).

The production and commercialization of cannabis products by this type of producers are not legal yet. According to the interviews collected, even though one aim of the 2020 law is to provide a legal framework for informal producers to register their products as “plant products” in the MSP, this has not been implemented yet. Even more, obtaining this permission might not be as easy for producers as it seems. The requirements for “plant products” of any kind stipulated in the Decree 403/016 (Presidencia de la República 2014) include, for instance, published evidence and technical documentation of the product’s use, details about the conditions for its indication, and its potential risks, among others. From the producers’ perspective, regulation is observed positively, as it would grant them a legal basis for their work. However, they also recognize that becoming a legal alternative has important implications for their procedures and practices: technical professionalization of human resources, better infrastructure, testing, and analysis of their products, among others. Of course, these would imply higher costs, something that not all might be willing, or be able, to face.

On the other hand, a big concern for producers’ associations is the presence of new unregulated sellers that offer low-quality products in street fairs or online. Pablo Silva, a member of the Cannabis Oil Users Association, described in a Deputy’s committee: “Today anyone can access cannabis oil. That is why we want a regulated alternative to be accessible. We come here to be controlled; we want to be controlled so people can access what they really want and not a fraud” (Comision Especial de Adicciones 2017). Becoming legal seems to be the answer for them, as it represents a way of ensuring the quality of the products offered. This concern is shared by health professionals that have prescribed cannabis and observe the need for products that ensure systematization in the preparation procedures, evidence on their efficacy and safety, and medical follow-up.

## Discussion

Uruguay approved the medicinal and recreational uses of cannabis simultaneously, becoming a pioneer in the drug-policy field. However, the recreational market was implemented faster than the medicinal one. In its origins, regulation was designed mainly with a public security objective (Queirolo et al. 2019). In order to remove users from criminal environments, the normative introduced three legal mechanisms of access for recreational purposes: homegrown, cannabis social clubs, and dispensation through pharmacies. Since 2017, the three options have been fully operative, and by 2021, almost seventy thousand users have registered to access recreational cannabis legally. In contrast, the medicinal use, and the development of a national industry around it, have not been at the center of the public policy discussion. Consequently, after almost a decade of its approval, the medicinal cannabis policy has remained halfway to guarantee access to patients or promote an appealing economic opportunity. Furthermore, the regulation implemented can be described as one that aims for quality and is highly state-centered, what has directly impacted patients’ access.

After conducting twelve in-depth interviews with relevant stakeholders, we identify three relevant areas in which key challenges persist. First, we detect barriers to the growth of a cannabis-based industry. Although several licenses have been approved and the national government has pushed reforms to promote it, institutional tensions among the different agencies in charge of licensing companies and the overlapping of responsibilities remain. These controversies are notorious for companies’ representatives and entrepreneurs, who observed that the differences in administrative requirements frequently become an obstacle for them. On this issue, the recently approved repealing decree of 46/015 can be seen as a manifest attempt to overcome these tensions and reorganize the licensing process in favor of the industry.

Second, there are still significant challenges regarding patients’ access to cannabis-based products and treatments. The legal options available are scarce, expensive, and limited to some conditions. Accessibility issues increase, as cannabis-based medicines are not covered for their dispensation through health system’s institutions. The two legal alternatives are also problematic. On the one hand, the importation of medicines from other countries is not receiving new petitions. On the other hand, although allowed by the 2019 law, magistral preparations have not been regulated yet, meaning that this alternative is not a real option now. In short, we can affirm that currently, cannabis is a hard-to-reach option for many patients.

Finally and highly connected with the last challenge is the emergence of a growing unregulated market of small producers that gain ground among patients. This sector, principally organized in civil associations, operates at the margins of the regulation. A possibility might be to register their production as a “vegetable specialty” once the Executive regulates the 2019 law. However, this alternative presents challenges of its own. The requirements for that category in terms of quality checks, standardization of procedures, and reporting evidence might be too stringent for many of them. Nevertheless, testimonies collected for this research show that producers intend to move towards a legal framework that protects their work. An interesting example in this subject is Colombia, whose medicinal cannabis regulation includes small-scale producers and forces industries to acquire raw materials from them (Aguilar et al. 2018).

This paper has several limitations. First, our design does not allow us to understand if the conflicting forces mentioned before are exclusive of the medicinal cannabis regulation. It is probable that the Uruguayan bureaucracy responds in the same way to other kinds of regulations, especially if they concern public health. In this article, we are unable to size that effect. In the same way, we cannot isolate the effect of Uruguay’s place in the international markets. This could partially explain the degree of development of the national cannabis industry. Because our focus is on the government institutions, we do not explore this issue. Further research needs to be done to explore both topics. Secondly, because we pursued interviewees who are directly related to the implementation of this policy, we did not include antagonist voices. This decision was made because of our specific interest is understanding how the policy is being implemented. Nonetheless, this could have resulted in an over-representation of these opinions, compared to more critical positions towards the regulatory process.

## Conclusion

The three challenges presented above are interconnected. Solutions implemented on one would have direct consequences on the others. For instance, if the recently approved normative accelerates the national cannabis-based industry, it is expected to generate a more diverse supply at pharmacies and, consequently, more options for patients. The same logic operates if Law 19.847 is finally regulated and implemented. As we mentioned, the legal basis of the medicinal cannabis policy is still under review. In addition, the interviews showed that authorities, entrepreneurs, and small producers are very aware of the extent of these challenges.

Until now, mechanisms to access legal recreational cannabis―home-growing, cannabis clubs, and dispensation through pharmacies―are not available for medicinal uses, so they have not been considered as a shortcut to expand access to patients. However, in reality, many home-growers cultivate for medicinal purposes (Queirolo et al. 2021) and clubs have medicinal users as members (Pardal et al. 2019). The paradox is that while these mechanisms are forbidden to be used for medicinal purposes in order to preserve quality-controlled products, they are already being used unregularly. The pursuit of patient security is, from this point of view, being backfired.

We began this article by stating that Uruguay is well known for its cannabis policy. However, the important challenges that the medicinal component still faces have garnered less international attention. Although the regulation was approved years before many other countries, patients who use cannabis for medical conditions continue to deal with a poor legal market and a growing unauthorized supply. In addition, the industry continues to cope with uncoordinated governmental agencies. In conclusion, despite the country early regulation, medicinal cannabis policy faces conflicting forces and challenges in its implementation.

## Supplementary information

**Additional file 1.** Interviews Guide.

## Data Availability

Not applicable.

## Abbreviations

IRCCA: Instituto de Regulación y Control del Cannabis (Cannabis Regulation and Control Institute)
MSP: Ministerio de Salud Pública (Ministry of Public Health)
MGAP: Ministerio de Ganadería, Agricultura y Perca (Ministry of Livestock, Agriculture and Fishing)
FTM: Formulario Terapéutico de Medicamentos (Medicaments Therapeutic Form)
