# Supplementary material for: Conflicting forces in the implementation of medicinal cannabis regulation in Uruguay
Source: J Cannabis Res. 2023 Jul 12;5:26. doi: 10.1186/s42238-023-00189-6 (PMC10337049; doi:10.1186/s42238-023-00189-6)
Supplement: Supplementary file 1 — Additional file 1. Interviews Guide. [file 42238_2023_189_MOESM1_ESM.pdf]

Additional file 1 — Interviews Guide

This is the questionnaire used for the interviews.

About the normative:

1. How do you see the current situation of the regulation regarding access to medicinal or therapeutic cannabis?
2. In your opinion, what are the most significant changes introduced by the law approved in 2020?

About patients' access:

1. What is the current situation of patients' access to medicinal cannabis products? What products are available? What would you say are the most important barriers that users have today in accessing products?
2. And concerning the creams, oils, and other products that medicinal users usually use, what are the main barriers to accessing these products? Has current legislation improved access?
3. Thinking about small producers, what are their current situation and their main challenges?

About physicians:

1. How would you describe the medical community's position regarding medicinal cannabis, either to prescribe it or to recommend its use? Are there more "open" medical specialties?
2. Among those who still do not agree on its prescription, what would you say are the main objections they express to do so?
3. In scientific terms, what is the most important current evidence on the effects of medical cannabis? For what types of patients or for what conditions? With what side effects?
4. International experience has found that the lack of instruction during the training of doctors -at university- is one of the main barriers that professionals identify for the prescription of medical cannabis. What happens in Uruguay? Is there training?
5. Has there been interest from universities in including the topic? Do you think that if it were included in the career curriculum, it would be a boost for the acceptance of physicians?

About the industry:

1. How has the cannabis-based medicine industry evolved in Uruguay?
2. The government has expressed its intention to promote the cannabis industry. What are the main barriers you are currently facing?
3. How is the relationship between the MSP and the IRCCA? Is obtaining licenses/authorizations easy?
4. How has the medical cannabis market received Uruguayan products?
5. How has been the acceptance of the physicians of the medicinal products made in Uruguay?
